# The Potential Role of Cyclopeptides from *Pseudostellaria heterophylla*, *Linum usitatissimum* and *Drymaria diandra*, and Peptides Derived from Heterophyllin B as Dipeptidyl Peptidase IV Inhibitors for the Treatment of Type 2 Diabetes: An In Silico Study

**DOI:** 10.3390/metabo12050387

**Published:** 2022-04-24

**Authors:** Hui-Jun Liao, Jason T. C. Tzen

**Affiliations:** Graduate Institute of Biotechnology, National Chung Hsing University, Taichung 402202, Taiwan; tctzen@dragon.nchu.edu.tw

**Keywords:** *Pseudostellaria heterophylla*, *Linum usitatissimum*, *Drymaria diandra*, cyclic peptides, cyclopeptides, DPP4, Heterophyllin B, Cyclolinopeptide, Diandrine C, diabetes

## Abstract

Dipeptidyl peptidase 4 (DPP4) inhibitors can treat type 2 diabetes by slowing GLP-1 degradation to increase insulin secretion. Studies have reported that *Pseudostellaria heterophylla*, *Linum usita-tissimum* (flaxseed), and *Drymaria diandra*, plants rich in Caryophyllaceae-type cyclopeptides and commonly used as herbal or dietary supplements, are effective in controlling blood sugar. The active site of DPP4 is in a cavity large enough to accommodate their cyclopeptides. Molecular modeling by AutoDock Vina reveals that certain cyclopeptides in these plants have the potential for DPP4 inhibition. In particular, “Heterophyllin B” from *P. heterophylla*, “Cyclolinopeptide C” from flaxseed, and “Diandrine C” from *D. diandra*, with binding affinities of −10.4, −10.0, and −10.7 kcal/mol, are promising. Docking suggests that DPP4 inhibition may be one of the reasons why these three plants are beneficial for lowering blood sugar. Because many protein hydrolysates have shown the effect of DPP4 inhibition, a series of peptides derived from Heterophyllin B precursor “IFGGLPPP” were included in the study. It was observed that IFWPPP (−10.5 kcal/mol), IFGGWPPP (−11.4 kcal/mol), and IFGWPPP (−12.0 kcal/mol) showed good binding affinity and interaction for DPP4. Various IFGGLPPP derivatives have the potential to serve as scaffolds for the design of novel DPP4 inhibitors.

## 1. Introduction

### 1.1. About Diabetes

Diabetes is a metabolic disorder characterized by increased blood glucose levels. With urbanization and social and cultural change in food behavior, the prevalence of diabetes is rapidly increasing. Diabetes and its extended vascular complications, diabetic nephropathy, retinopathy, etc., cause heavy physical and mental pressure to patients [1]. Diabetes is classically divided into three types: type 1, type 2, and gestational diabetes, with type 2 diabetes (T2D) accounting for the majority. Type 2 diabetes is caused by insulin resistance, which refers to impaired sensitivity to insulin-mediated glucose disposal or insufficient insulin secretion [2]. Type 2 diabetes can be prevented by maintaining a normal weight, regular exercise, and eating a healthy diet [3]. However, some will still face the need for medication. Metformin is the first-line hypoglycemic agent recommended when most patients are initially on drug control. In addition to metformin, new hypoglycemia treatments are constantly being proposed due to the high demand for T2D drugs and the emergence of resistance.

### 1.2. Mechanisms of Incretins (GLP-1 and GIP) in Glucose Homeostasis and Diabetes Treatment

Incretins are hormones secreted by endocrine cells of the intestinal epithelium with food to maintain blood sugar balance. The two most studied incretins are glucagon-like peptide 1 (GLP-1) and glucose-dependent insulinotropic polypeptide (GIP) [4]. Studies have found that GLP-1 and GIP can stimulate pancreatic β-cells to increase insulin synthesis and secretion to help stabilize blood sugar after meals [5]. More follow-up studies have indicated that GLP-1 has a greater impact on blood sugar than GIP. In addition to stimulating insulin secretion from β cells, GLP-1 can also inhibit the secretion of glucagon from α cells and increase the secretion of somatostatin by δ cells. Therefore, GLP-1 accounts for more than GIP in studies of diabetes based on the incretin system [6,7]. The bioactive forms of GLP-1 include GLP-1 (7−37) and GLP-1 (7−36) NH_2_. These active peptides maintain blood glucose homeostasis by activating the GLP-1 receptor (GLP-1R) on β cells, triggering a series of downstream reactions. The activation of GLP-1R by GLP-1 or GLP-1 analogs directly results in cAMP accumulation, followed by increased insulin secretion. Longer-term effects also include promoting β-cell proliferation and reducing β-cell apoptosis. This is of great significance in delaying the depletion of pancreatic islet cells in diabetic patients [6,7]. The effects of GLP-1 are also involved in extra-pancreatic events. The topic of greatest interest was the effect of GLP-1 on appetite and weight loss. The release of GLP-1 and related effects delay gastric emptying and bowel motility, and in addition, exert pressure on the hypothalamus to alter satiety; thereby, suppressing appetite and assisting in weight control [7].

Endogenous GLP-1 in the circulation will be immediately degraded by dipeptidyl peptidase 4 (DPP4) into inactive metabolites GLP-1 (9−37) and GLP-1 (9−36) NH_2_ (t 1/2 ~1–2 min). In T2D patients, reduced GLP-1 secretion or decreased GLP-1 response, also known as incretin deficiency, has been observed, resulting in poor postprandial blood glucose regulation [6]. DPP4 inhibitors are designed to interfere with the enzymatic activity of DPP4, reducing its rate of cleavage of GLP-1 to increase the concentration of active GLP-1 in plasma. Another incretin-based therapy for T2D is to apply GLP-1 analogs to mimic the effects of endogenous GLP-1. The hypoglycemic effects of incretin-based therapies for T2D have been proven [7]. Clinically available oral DPP4 inhibitors include Sitagliptin, Vildagliptin, Saxagliptin, Alogliptin, Linagliptin, etc. GLP-1 analogs such as Exenatide, Liraglutide, and Semaglutide (now also available in oral form) typically require administration by injection and are therefore less convenient than the orally available DPP4 inhibitors [7,8,9]. One of the advantages of incretin-based therapies is that since the effect of GLP-1 depends on blood glucose concentration, it rarely causes hypoglycemia. After the treatment reaches the tolerated dose of metformin and the patient’s blood sugar control is still unsatisfactory, DPP4 inhibitors can be used alone or in combination with metformin as second-line therapy [10]. According to clinical observations, DPP4 inhibitors have an apparent influence on the activity of DPP4, reducing the baseline by more than 50% [11]. When Sitagliptin (the first listed DPP4 inhibitor) monotherapy is used to treat adult T2D patients, the observed efficacy includes improvement in HbA1c, fasting plasma glucose (FPG), and 2-h postprandial glucose (PPG). In addition, most studies have reported its beneficial effects on the regulation of triglyceride (TG), HDL-c, and LDL-c [6,12]. 

### 1.3. The Structure of DPP4 and the Interaction of DPP4 Inhibitors with DPP4

**Figure 1:**
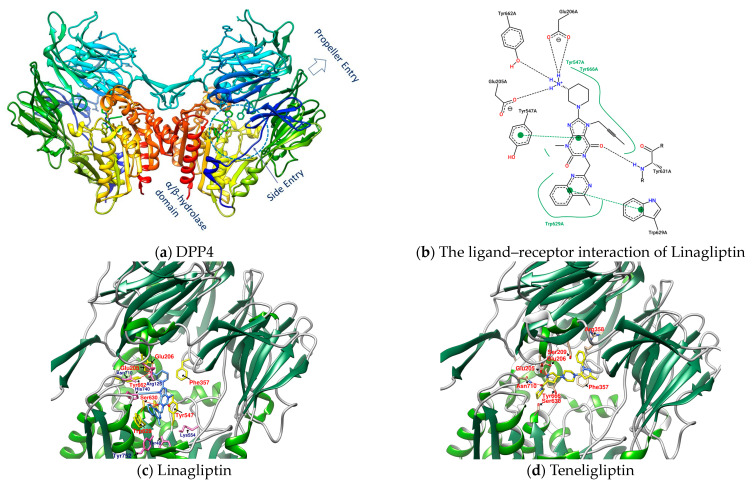
(**a**) DPP4 (PDB: 2RGU); (**b**) Linagliptin’s PoseView analysis; (**c**) Linagliptin (PDB: 2RGU) and (**d**) Teregliliptin (PDB: 3VJK) rendered by UCSF Chimera (red lines are hydrogen bonds).

Dipeptidyl peptidase 4 (DPP4), also known as CD26 (cluster of differentiation 26), is a type II transmembrane serine protease with 766 amino acids (110 kDa) anchored to the membrane, that selectively cleaves the Xaa-proline or Xaa-alanine dipeptides from the N-terminus of GLP-1 [11]. Transmembrane DPP4 enhances its enzymatic activity through dimerization. Matrix metalloproteinases (MMPs) cleave DPP4 on the membrane. Cleaved DPP4 lacks the cytoplasmic domain (aa 1-6), transmembrane domain (aa 7-28), and flexible stalk (aa 29-39) and becomes a circulating or soluble form (sDPP4, aa 39-766) [11,13,14,15]. sDPP4 is less studied relative to DPP4 on the membranes. The extracellular part of the monomer in dimeric DPP4, in addition to the flexible stalk, mainly includes a large cavity constructed between the eight-bladed β-propeller domain (aa 54-497) and α/β hydrolase domain (aa 39-51 and 506-766) with a set of entrances for GPL-1 and GIP, etc. to in and out [11,13,14,15]. The catalytic triad (composed of Ser630, Asp708, and His740) and its adjacent amino acids Glu205 and Glu206 (to ensure the anchoring of the N-terminus of the substrate) have a great influence on the enzymatic activity of DPP4. Moreover, Arg125 and Asn710 contribute to electrostatic adsorption; Tyr662 and Tyr666 form hydrophobic pockets; and Tyr547 is responsible for oxygen anion holes, constituting a series of important amino acids in the active site of DPP4 [11]. The area enclosed by the amino acid residues Glu205, Glu206, Tyr662, Ser630, Trp629, and Tyr547 is the site where Linagliptin (PDB: 2RGU) and most DPP4 inhibitors are located (Figure 1) [16]. 

**Figure 2:**
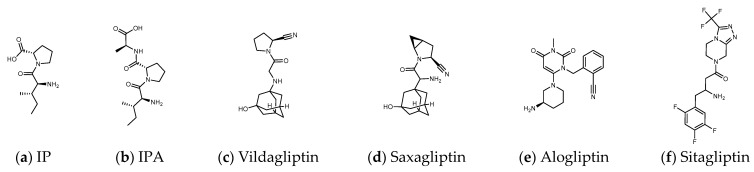
(**a**,**b**) Peptide DPP4 inhibitors. (**c**–**f**) Clinical DPP4 drugs. Compared with Sitagliptin, the molecular size and proline-contained structure of the dipeptides IP and IPA are closer to Vildagliptin and Saxagliptin. However, Vildagliptin and Saxagliptin are cyanopyrrolidine-bearing compounds that can form a covalent bond with DPP4. In order to establish more hydrogen bonds between the peptide and DPP4, it may be necessary to increase the length of the sequence.

Some inhibitors may also expand the discussion to the outer region formed by Arg125, Asn710, His740, Tyr752, Tyr48, and Lys554. There is also a design strategy for DPP4 inhibitors, in which the inhibitor approaches Lys554 in the S1′ pocket to form a salt bridge and establishes a hydrophobic interaction with Tyr547 to achieve the effect instead of binding to the amino acid residues in the catalytic center [17]. Additionally, it was observed that the (1-phenylpyrazol-5-yl) piperazine moiety of Teneligliptin (PDB: 3VJK) extends to Ser209, Phe357, and Arg358 closer to the β-propeller domain [18]. Vildagliptin (PDB: 6B1E) and Saxagliptin (PDB: 3BJM) are two cyanopyrrolidine-bearing compounds with smaller molecules than Linagliptin. The main structure of their crystals in DPP4 only occupies the more concentrated area between “Glu205, Glu206, and Ser 630” and forms a covalent bond with Ser 630 (Figure 2) [19].

### 1.4. Natural Products with Relevant Reports on Lowering Blood Sugar and Their Mechanisms 

Many herbs have been shown to have the effect of regulating blood sugar and have become a choice of dietary supplements for patients with T2D. Studies have found that DPP4, PTP1B, α-glucosidase, AMPK, PPARγ, etc. are the targets that natural products may be involved in hypoglycemia [20]. These hypoglycemic natural substances include a large number of phenols, lignans, terpenes, alkaloids, protein hydrolysates, etc., but cyclic peptides (cyclopeptides) are still rare. Due to the highly charged nature of the catalytic domain of PTP1B, the oral drug design of PTP1B inhibitors remains a very challenging task [21]. Inhibition of α-glucosidase reduces the intestinal absorption of glucose and slows postprandial blood glucose rise, which is the possible hypoglycemic mechanism of many natural products [22]. However, if drug intake increases pancreatic β-cell density and improves fasting blood glucose, the pharmacological mechanism may go beyond the inhibition of α-glucosidase. DPP4 inhibition is another hypoglycemic target that may be involved. The triterpenoids quinovic acid-3β-O-β-d-glycopyranoside, lupeol, and phytosterol stigmasterol isolated from natural anti-diabetic plant *Fagonia cretica* L. and *Hedera nepalensis K. Koch* have been demonstrated to have inhibitory effects on DPP4 [23]. Flavonoids and phenols such as luteolin, apigenin, quercetin, isoquercetin, rosmarinic acid, naringin, and eriocitrin also revealed the efficacy in inhibiting the activity of DPP4 [24]. Curcumin is evaluated as an α-glucosidase and DPP4 inhibitor, and is recommended for the management of diet-induced hyperglycemia [25]. In addition, many protein hydrolysates from natural resources show potential as DPP4 inhibitors [26,27] such as LKPTPEGDL and LKPTPEGDLEIL from pepsin-treated bovine whey proteins [28]; LPQNIPPL from gouda-type cheese [29]; PPPP, GP, PP, MP, VA, MA, KA, LA, FA, AP, FP, PA, LP, VP, LL, VV, HA, IPA, and IPI from the hydrolysis of amaranth proteins [30]; and LP and IP from defatted rice bran (Figure 2) [31]. The molecular weights of these peptides vary widely. Although the spacing of Phe357, Ser209, Glu205, and Ser630 to Lys554 in DPP4 is sufficient to accommodate large molecules, small molecules can also occupy the vicinity of the catalytic center and hinder enzyme activity. This may explain why LKPTPEGDLEIL and IP both show the bioactivity of DPP4 inhibition in related studies.

### 1.5. Pseudostellaria Heterophylla, a Reported Natural Product with Hypoglycemic Effect 

**Figure 3:**
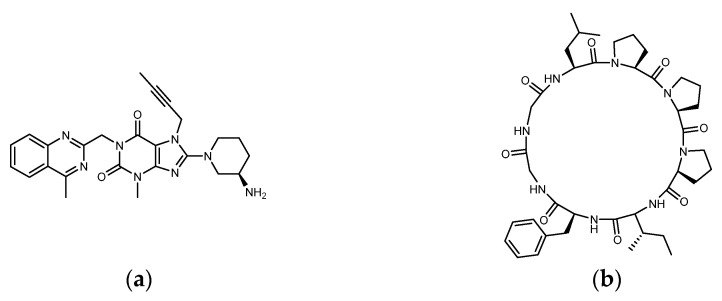
Structure of (**a**) Linagliptin and (**b**) Heterophyllin B.

*Pseudostellaria heterophylla* (Heterophylly Falsesatarwort Root, Taizishen, or *P. hetero-phylla*) is rich in cyclic peptides (cyclopeptides) and is reported to be a medicinal plant with hypoglycemic effect. *P. heterophylla*, belonging to the Caryophyllaceae family, is known as the “ginseng of the lungs” (similar to ginseng that is good for the lungs). According to the herbal pharmacopoeia record, it is suitable for improving dry cough, loss of appetite, fatigue, mental exhaustion, and physical weakness after illness. It is also used as a nutritional supplement for children with a weak physique. In modern times, *P. heterophylla* is one of the important materials used in clinical Chinese compound prescriptions for improving hyperglycemia. It is rich in polysaccharides, saponins, and cyclopeptides, among which Heterophyllin B (HB) is one of its quality indicators [32,33,34]. Studies have already shown that *P. heterophylla’s* polysaccharides and saponins have the effect of lowering blood sugar [35,36]. However, a review of the hypoglycemic effect of its cyclopeptides is lacking. Heterophyllin A (HA) and HB were found in 1991 as the first cyclopeptides identified from *P. heterophylla* and encouraged a large number of studies on cyclopeptides in the next 30 years [37]. In the classification of cyclopeptides by NH Tan et al., the cyclopeptides from *P. heterophylla* including Heterophyllin A, B, C, J, and Pseudostellarin A~H were entered into the category of “Caryophyllaceae-type cyclic peptides” (CTCs) (CTCs: homo-mono-cyclopeptides formed with the peptide bonds, which include cyclic dipeptides to dodecapeptides) [38]. In 2013, PG Arnison et al.’s recommendations for a universal nomenclature suggested that plant N–C cyclic peptides lacking disulfide bonds and significantly biased toward hydrophobic amino acids could be classified as “Orbitides”. The cyclic peptides listed in CTCs and Orbitides are approximately the same, involving at least nine independent plant families. In addition to Caryophyllaceae, it also includes Annonaceae, Linaceae, and Rutaceae, etc. Some non-Caryophyllaceae-derived cyclic peptides used to be called Orbitides such as those derived from flaxseed. Studies have shown that Orbitides have many biological activities including cytotoxicity, antiplatelet, antimalarial, immune regulation, immune suppression, etc. [39]. Recently, Feng Lu et al. found that *P. heterophylla’s* cyclopeptides can ameliorate COPD (chronic obstructive pulmonary disease) and reduces lung inflammation via the TLR4/MyD88 pathway; moreover, the 28-days animal test of 500 mg/kg purified extract (by oral administration) showed no toxicity [40]. Recently, in related studies on DPP4 as a therapeutic target for lung diseases, it was found that DPP4 may be involved in the pathophysiology of COPD [41]. Moreover, DPP4 inhibition by Sitagliptin was found to be able to attenuate LPS-induced lung injury in mice [42]. Is it possible that the effect of *P. heterophylla’s* cyclopeptides on COPD is related to the inhibition of DPP4? In addition to polysaccharides and saponins, does the indicator compound HB participate in the hypoglycemic mechanism? It can be observed that the diameter of HB (octacyclic peptide) is close to the length of Linagliptin (Figure 3). Could parts of the HB ring be used to match or be close to the region in DPP4 where Linagliptin acts? Since natural ligands of DPP4 such as GLP-1 and GIP are peptides, there has been much discussion of whether protein hydrolysates are involved in DPP4 inhibition. Is it possible that the cyclopeptides of *P. heterophylla* are involved in the DPP4 inhibition similar to some protein hydrolysates?

Many natural proline-rich cyclopeptides from marine organisms have been found to be very similar in appearance to plant-derived Orbitides, but their structure may be interspersed with non-peptide elements [43]. There have been many physiological and pharmacological studies on marine cyclopeptides including anti-fertility, anti-cancer, anti-viral, etc. Many sponge-derived cyclopeptides such as the “Phakellistatin 1–19” series are well-known cytostatic compounds for the development of anticancer agents. In the “structure–activity relationship” study of Phakellistatins, it was found that because proline residues can reduce the flexibility of the backbone, the proline-rich cyclopeptides can enhance the selectivity and affinity of their receptors [43,44,45]. The health-oriented hydrolyzed dairy products also emphasize the role of their proline-rich peptides. The proline-rich polypeptide complex Colostrinin™, isolated from ovine colostrum, has immunoregulatory properties and shows beneficial effects on neurodegenerative diseases [46]. In addition, the aforementioned protein hydrolysates PP, PPPP, IP, and LPQNIPPL were reported to have an effect on DPP4 inhibition. The peptide sequence IFGGLPPP of Heterophyllin B is also enriched in proline. With a large number of plant-derived cyclopeptides, proline-rich may be an option to narrow down the search when screening for specific cyclopeptides of interest. Among the existing peptide drugs, cyclopeptides account for the majority due to their higher lipophilicity, higher membrane permeability, in vivo stability, and higher specificity for target receptors [47,48]. Plant cyclopeptides are currently receiving attention in many aspects such as anti-tumor, immune regulation, sedation, antibacterial, antiviral, and so on [38,39]. However, there are relatively few reports on the use of cyclopeptides for lowering blood sugar. Therapeutic drugs or dietary supplements for patients with type 2 diabetes need to consider the safety of long-term use, so it is necessary to avoid toxic species. Many marine cyclopeptides and disulfide-rich cyclotides are cytotoxic and are not suitable for development as dietary supplements. Besides, the possible changes in the configuration of cyclic peptides composed of disulfide bonds are much more complex relative to “Caryophyllaceae-Type Cyclopeptides”. Is there a chance to find other natural substances with hypoglycemic reports in the group containing CTCs, in addition to *P. heterophylla*? In this context, *Linum usitatissimum* (flaxseed) and *Drymaria diandra*, which are rich in CTCs, have been examined and compared with *P. heterophylla* under the hypoglycemic theme. They have many pharmacological effects including lowering blood sugar, and are documented for nutrition or daily health care. In addition, many of their cyclopeptides contain two proline residues [38]. 

### 1.6. Linum Usitatissimum, Which Is Rich in Cyclic Peptides and Has Hypoglycemic Effect Reported

*Linum usitatissimum* (flax, flaxseed, or linseed) contains α-linoleic acid, lignans, and cyclic peptides, etc., and is mainly used as flaxseed oil and dietary flaxseed meal. Many flaxseed studies take the term “Orbitides” to refer to its cyclopeptides. As of 2019, 39 flaxseed cyclopeptides have been isolated from flaxseed oil with a high content (more than 100 mg/100 g) [49]. According to the open-label study by Mani, U.V. et al., by supplementing 10 g of flax seed powder (FS) daily for 1 month and keeping drug intake unchanged, reductions in fasting blood glucose (FBG), glycated hemoglobin, total cholesterol, and triglyceride values were observed in the experimental group [50]. The hypoglycemic effects of flaxseed lignans have been reported in the nutrition literature. A study by A Pan et al. showed that flaxseed-derived lignan supplement improved HbA1c, but no significant difference was observed in fasting plasma glucose (FPG), insulin concentration, insulin resistance, and lipid profile [51]. There were differences in the results of hypoglycemic observations on flax lignans and FS, although the background of the experiment was not exactly the same. This led to consideration of whether there are ingredients other than lignans in flaxseed that are involved in the blood sugar-lowering mechanism and may have a broader impact. A de novo peptide sequencing study by the *CycloNovo* analysis method revealed that many flaxseed cyclopeptides in the human gut such as Cyclolinopeptide A, B, D, E, H, etc. [52]. The biological activity of the cyclopeptide further provides support for thinking about whether flaxseed Orbitides can reduce the enzyme activity of DPP4 and participate in the hypoglycemic mechanism.

### 1.7. Drymaria Diandra, Which Is Rich in Cyclic Peptides and Has Hypoglycemic Effect Reported

*Drymaria diandra* (*D. diandra*, *Drymaria cordata Willd*, or *D. cordata*), also known as tropical chickweed, belongs to the family Caryophyllaceae. It grows quickly in some humid and warm places in Africa, Asia, and the Americas and used as a folk medicine for anti-inflammatory, antibacterial, antipyretic, analgesic, and acute hepatitis [53,54,55]. Similar to *P.* *heterophylla*, *D. diandra* has also been reported as an antitussive effect. People in some areas use it when they have a cold or cough [56]. The main pharmacological ingredients of *D. diandra* are cyclopeptides, flavonoids, and alkaloids. *D. diandra* leaves can be sun-dried and boiled into herbal tea. In addition, it is said that the fresh leaves can be ground lightly and applied to the wound or diluted with honey water to treat fever [55,57,58]. Compared with the obvious symptoms of inflammation and fever, diabetes is a new concept for traditional medicine. Even in modern times, a considerable proportion of people do not know that they have diabetes. Therefore, it is not easy to find the “hypoglycemic” term in the local herbal pharmacopoeia. The ethnic groups of the Sikkim in India use *D. diandra* for various diseases including diabetes. *D. diandra* has become one of the few regional herbal medicines that can enter the field of contemporary diabetes research. S Patra et al. tried to treat diabetic rats with a *D. diandra* (*D. cordata*) methanol extract (DCME) to observe changes in various physiological indicators. DCME is still safe at an oral dose of 2000 mg/kg. Compared with the diabetes group, HbA1c, FBG, and lipid profiles in the DCME group were reduced, and the β cell density was improved in a dose-dependent manner. Their studies speculated that the α-glucosidase inhibitory activity of DCME and the antioxidant properties of flavonoids and alkaloids are responsible for the improvement of type 2 diabetes [59]. Since experimental values overlap with the effect of DPP4 inhibition, and the cyclopeptides “Diandrine A–D” are present in the methanol extract according to the early identification by PW Hsieh et al., is it possible that cyclopeptides from *D. diandra* are also involved in the hypoglycemic mechanism based on incretin [57]?

### 1.8. Can Linear Precursors of Heterophyllin B “IFGGLPPP” Participate in DPP4 Inhibition?

Numerous protein hydrolysates have shown the effect of DPP4 inhibition. However, the process of identifying functional peptide sequences from hydrolysis, isolation, purification, and bioassay to mass spectrometry analysis is difficult and time-consuming. Two linear precursors of Heterophyllin B (HB), “GGLPPPIF”, and “IFGGLPPP” have been previously reported. The sequence of IFGGLPPP was verified by precursor gene (prePhHB) screening and in vitro and in vivo experiments, which were suggested to be more likely to be the precursor peptide of HB, later [33]. Is it possible that the linear peptide IFGGLPPP has a favorable binding affinity for DPP4? Additionally, can IFGGLPPP be modified to obtain more samples for comparative studies? For example, inserting other fragments such as PPPP, FP, WP, and PY into existing linear peptides, or changing the sequence of local fragments. DPP4 cleaves dipeptides such as Xaa-Proline or Xaa-Alanine (also includes Xaa-Gly, Xaa-Ser, Xaa-Val, etc., but mainly Xaa-Pro) from the N-terminus of the polypeptide, where Xaa stands for any amino acid. The sequence to be cleaved by DPP4 can be any amino acid except Proline at the third position [11]. Cleavage by DPP4 should be avoided when designing or developing linear peptides as DPP4 inhibitors. Since the samples to be explored contain a large number of cyclopeptides from *P. heterophylla*, flaxseed, and *D. diandra* as well as a series of IFGGLPPP-derived peptides, molecular modeling provides a feasible method for preliminary screening of potential cases. In the future, molecules with good DPP4 binding affinity screened by docking can be isolated, purified, or synthesized for more in vitro or in vivo studies.

## 2. Results and Discussion

### 2.1. The Binding Affinity of Three Plant-Derived Cyclopeptides to DPP4 and Their Research Potential

The natural substances *P. heterophylla*, flaxseed, and *D. diandra* are rich in cyclic 5–9 peptides, and related studies have reported their effects on lowering blood sugar. Regarding their possible hypoglycemic composition and mechanism, there is still a lack of research on cyclopeptides. Since cyclopeptide is an important component in these three plants, it may have an impact on the hypoglycemic effect. Considering that the catalytic center of DPP4 is located in a relatively large space, it may have a better chance of accommodating cyclopeptides than PTP1B, α-glucosidase, AMPK, and PPARγ (active site is smaller than DPP4). After docking Heterophyllin B (HB): (cyclo)-GGLPPPIF-(cyclo) with DPP4 (PDB: 3G0B) [60], the results showed that it can appear close to the place occupied by Linagliptin with favorable binding energy. The results of docking HB with DPP4 support the idea of extending the exploration to more cyclopeptides from *P. heterophylla*, flaxseed, and *D. diandra*. The relevant cyclopeptide sequences, abbreviations, and binding affinities involved in this docking study are shown in Table 1, Table 2 and Table 3 (in addition to drawing the HA structure separately, the initial structure files were downloaded from PubChem). The study of AM Bower et al. used AutoDock Vina to calculate the binding affinity of herbal components and provided in vitro IC_50_ data for DPP4 inhibition, which can be used as a reference value at this time: Hispidulin (−9.4 kcal/mol), IC_50_ = 0.49 (μM); Eriodictyol (−8.9 kcal/mol), IC_50_ = 10.9 (μM); and Sitagliptin (−9.6 kcal/mol), IC_50_ = 0.06 (μM) [61]. If these cyclopeptides can reach the docking value close to Eriodictyol, there may be a chance to achieve a certain effect on DPP4 activity with the dose of dietary supplement. Most of the cyclopeptides from the three plants can meet this standard.

In Table 1, HB (−10.4 kcal/mol) had the best binding affinity, followed by PB (−9.6 kcal/mol), PD, PH, PE, HA up to PA. The overall result showed their potential for DPP4 inhibition. The ingredients of *P. heterophylla* may vary slightly due to different strains, origins, and harvest seasons. HA and HB are the first two molecules whose structures have been determined in the series of *P. heterophylla* cyclopeptides [37]. HB is considered to be an indicator to check the quality standards of *P. heterophylla*, while PB is commercially available. The docking results of HB and PB with DPP4 accounted for the first and second positions in the *P. heterophylla* series, and they happened to be the most important components in *P. heterophylla*. The result suggests that DPP4 may be the target of these cyclopeptides, and may also explain why *P. heterophylla* is often used in Chinese medicine prescriptions for the treatment of hyperglycemia. The research of Feng Lu et al. has shown that the cyclopeptides of *P. heterophylla* have oral effectiveness and safety [40]. Alcoholic extracts of Radix *P. heterophylla* with cyclopeptides inside may have the opportunity to become a nutritional supplement for diabetic patients after more research. 

CLA in flaxseed was confirmed as early as 1959. In 1997, when H Morita et al. purified flaxseed (80% methanol extract), in addition to CLA, CLB was newly discovered (CLB accounts for about 0.0002%) [62]. CLC is the oxidized form of CLB. In Table 2, the performance of CLC, CLA, and CLB ranked the top three, and the binding affinity was −10.0, −9.8, and −9.8 kcal/mol, respectively. The binding affinity of most flaxseed Orbitides was better than −9.0 kcal/mol, which explains the possible hypoglycemic reason for supplementing with flaxseed. This also suggests DPP4 as one of the research targets of flaxseed Orbitides. Flaxseed is a grain, which is more conducive to promotion as a nutritional product. The oxidation of flaxseed oil occurs during the preservation process. Several flaxseed Orbitides contain Met residue. Oxidation of the sulfur atom on Met produces a series of derivatized Orbitides. Relative to the effect on oil quality, Met-oxidized Orbitides seem to have no negative impact on the inhibition of DPP4 (oxygen may increase H-bonding). 

When PW Hsieh et al. identified the components of *D. diandra*, the cyclopeptide with the highest content was DdC (0.0004% of the MeOH extraction of dry whole herbs) [57]. In the compositional analysis of *D. diandra* by Z Ding et al., DmA and DmB accounted for 0.00014% and 0.011%, respectively [55]. The content of DmB and DdC (hexapeptide) was higher. The binding affinities of DdC, DmB, and DmA to DPP4 were −10.7, −8.9, and −10.2 kcal/mol, respectively (Table 3). DdC had the most prominent binding affinity when docked to DPP4. The Sikkim area in India uses *D. cordata* to treat diabetes. S Patra et al. confirmed that the *D. cordata* MeOH extract can reduce blood sugar and improve the blood lipid index in diabetic rats [59]. *D. diandra’s* cyclopeptides are probably one of the influencing factors in its hypoglycemic effect. Inhibition of DPP4 may also help improve hyperlipidemia and control weight. This preliminary docking analysis of *D. diandra’s* cyclopeptides in DPP4 may lead to more research. In addition to lowering blood sugar, *D. diandra* may also be an option for dietary supplements for weight management later.

DdC contains “PYWP”; HB and DmA contain “PPP”; CLA, CLB, CLC, and PB contain “PPFF” or “PPF” residues. The docking results revealed that these proline-rich cyclopeptides appeared to have a better affinity for DPP4. Although the determination of binding affinity is mainly based on hydrogen bonding and π–π interactions, it cannot be directly inferred from the number of prolines contained. These cyclopeptides also happen to be the components that are easier to purify or have higher content in the original plant. In the process of biosynthesis, perhaps these proline-rich sequences are easier to cyclize through endogenous enzymes or less easily degraded. The docking suggests that DdC, HB, DmA, CLA, CLB, CLC, and PB have higher potential for further study.

### 2.2. Analysis of the Configuration and Conformation of Plant Cyclopeptides Docking with DPP4

**Figure 4:**
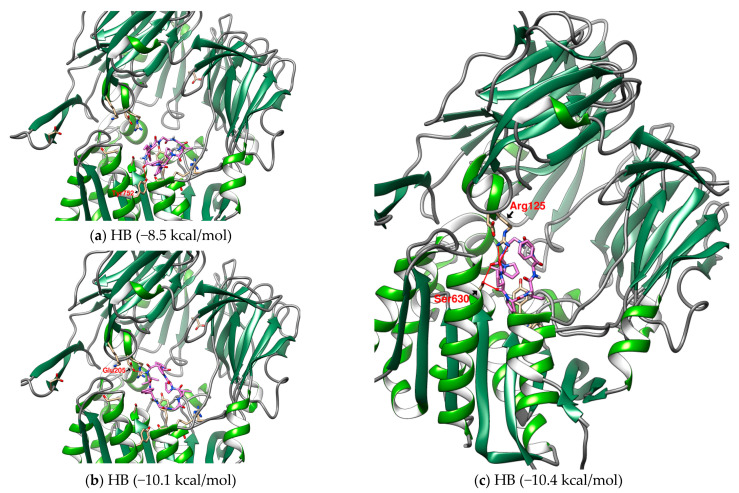

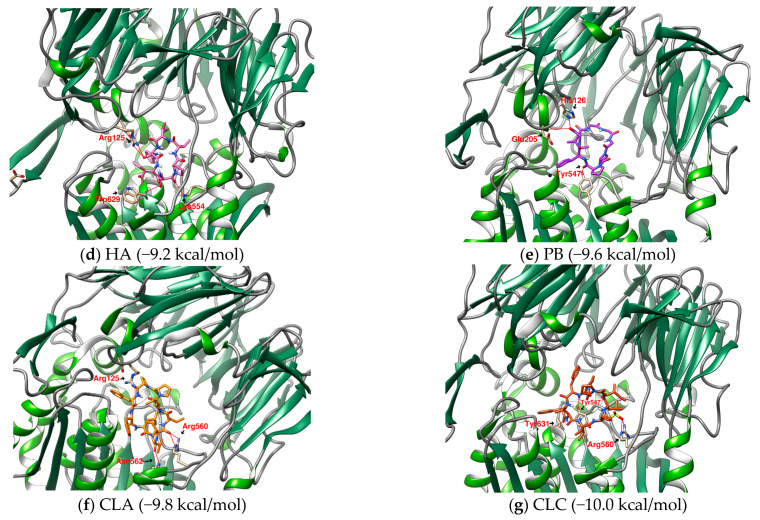
(**a**–**c**) The configurations of HB docked to DPP4 (three energy levels). (**d**–**g**) The lowest energy configuration for HA, PB, CLA, and CLC docked to DPP4 (binding affinity). Hydrogen bonds are shown as red lines and labeled with amino acid residues.

**Figure 5:**
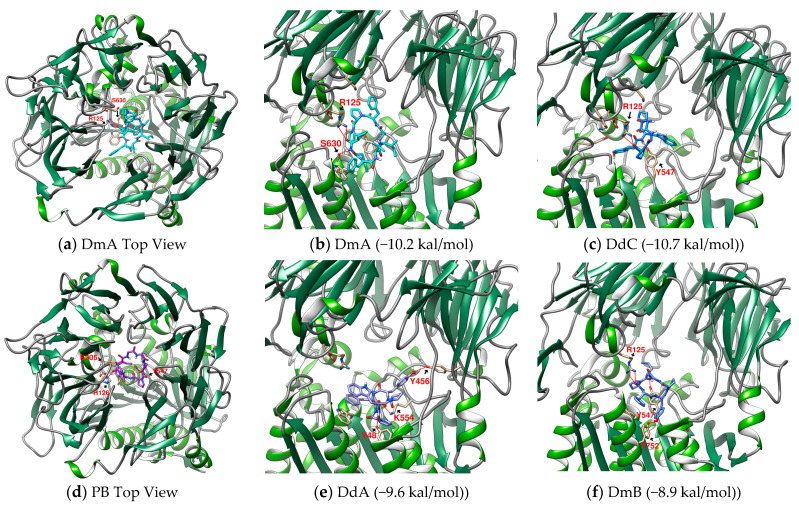
(**b**,**c**,**e**,**f**) Prediction of DmA, DdC, DdA, and DmB when docked with DPP4. (**a**,**d**) Top View of DmA and PB. Hydrogen bonds are shown as red lines and labeled with amino acid residues.

**Figure 6:**
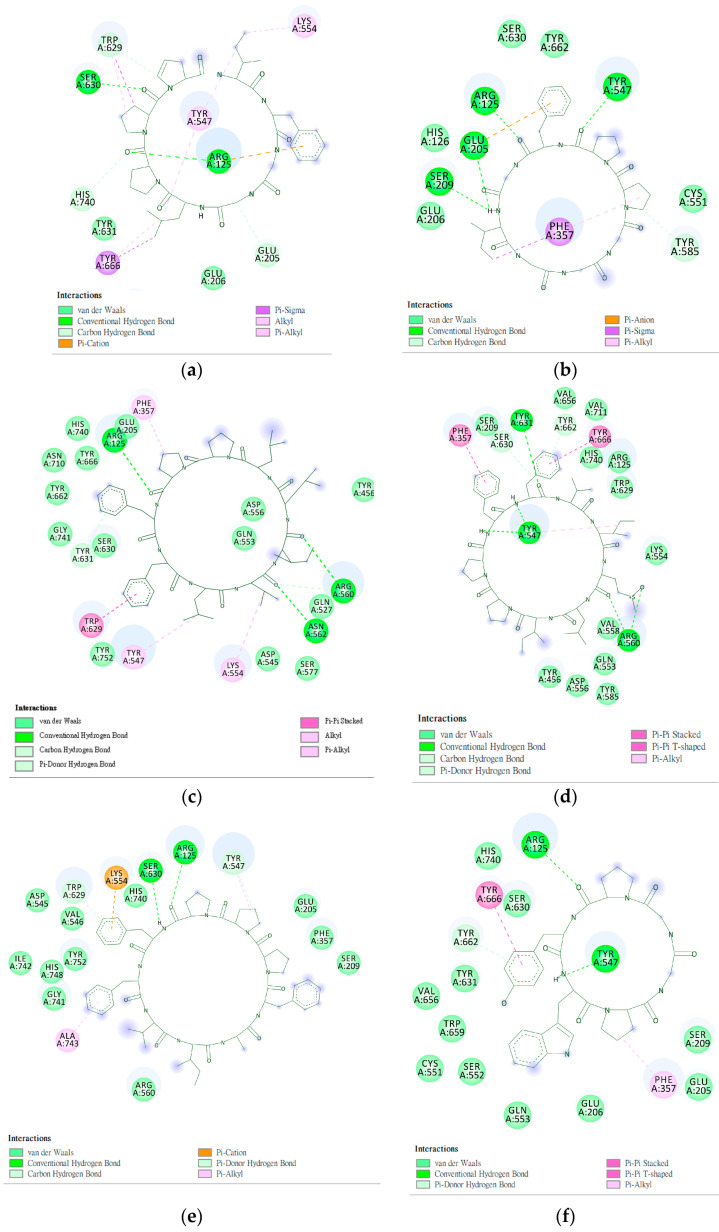
Interaction of DPP4 and ligands. (**a**–**f**) HB, PB, CLA, CLC, DmA, and DdC.

The interaction between a series of cyclopeptides and DPP4 is shown in Figure 4, Figure 5 and Figure 6. The main masses of HA, HB, DmA, and DdC appeared in the area surrounded by Arg125, Tyr547, and Ser630, similar to the place where Linagliptin is located. Although PB has hydrogen bonds with Tyr547 and Glu205, its position shifts to the entrance of the propeller. CLA and CLC are offset to the interval where Arg560 and Asn562 are sited. The “IFGGL” of HB constrains the ring and assists the three prolines to establish hydrogen bonds with Arg125 and Ser630, thereby blocking the entry of the catalytic triad. The Y and W of DdC are wrapped by flexible Gly and then by Pro (favorable to generate β-turns). Due to the hydrogen bond between PYWP and Arg125 andTyr547, plus the π–π interaction with Tyr666, it obtained a good binding score. The hexapeptides DdA and DmB had similar sequences to DdC but their interaction with DPP4 was not as good as DdC. DdC was located closest to the catalytic center, while DmB drifted toward Tyr752. The relationship between CLC and the catalytic region was weaker than that of DdC and HB. However, its hydrogen bond with Arg560 stabilizes the main structure and guides its Phe(F) to interact with Tyr666, which also has an impact on the catalytic activity of DPP4. The location of CLA, CLC may also have a negative impact on the entry of GLP-1. In contrast, the location of PB and DmA may interfere with the β-propeller region of DPP4 (top view).

Previously, in 2019, VCSR Chittepu et al. proposed a study on the DPP4 inhibition by the natural cyclic peptide oxytocin (IC_50_: 110.7 nM). Their molecular docking showed that oxytocin interacts with Arg 356, Phe 355, Tyr663, Glu 204, Glu 203, and Tyr548 [63]. Oxytocin research supports that the cyclic peptide may be a kind of DPP4 inhibitor. The oxytocin sequence is CYIQNCPLG-NH2, where CYIQNC forms a ring with disulfide bonds, and PLG is like a long side. Unlike oxytocin, cyclopeptides from *P. heterophylla*, flaxseed and *D. diandra* are plant-derived Orbitides without disulfide bonds; the longest side chains are only Met and Trp. Cyclic peptides without disulfide bonds can be calculated with the default setting such as general small molecules in AutoDock Vina. If the cyclic peptide contains disulfide bonds, the setting of the disulfide bonds and the interaction between the cyclic peptide and DPP4 may have more variables and uncertainties in the docking process. When the cyclic peptide does not have long branched side chains, the movements and rotations are less restricted. Such Orbitides can be rotated through various angles within the DPP4 cavity and finally placed in the most suitable location or close to the catalytic center of DPP4 with the lowest energy. These three types of plant-derived cyclopeptides are mainly composed of hydrophobic amino acids: AVILMFYW, plus Pro, and Gly. None of these plant-derived cyclopeptides have charged “RHKDE” amino acids. Since arriving at the active site of DPP4 means passing through the opening between the α/β-hydrolase and β-propeller domain, uncharged molecules may be able to avoid becoming stuck in the periphery due to the attraction of charges. 

The configuration of multiple energy levels of HB in the prediction may provide its possible movement in DPP4 (Figure 4). From the three predicted HB configurations with binding affinities of −8.5 kcal/mol, −10.1 kcal/mol, and −10.4 kcal/mol, it was found that as the affinity became stronger, the position of HB gradually approached the catalytic center. This may interfere with enzyme activity since HB drifts into DPP4, but eventually binds to the receptor in the conformation with the lowest binding affinity. In a series of research on marine cyclopeptides, it was observed that the rigidity of a proline-rich cyclic structure will reduce the entropy of the Gibbs free energy and enhance the binding force [43,44,45,64]. According to the observation of HB in DPP4, it was found that proline-rich fragments can provide multiple sites to establish hydrogen bonds in a small area. When this proline-rich fragment has the opportunity to be located near the catalytic center, it will increase the binding affinity, as shown by the case of the final stable conformation of HB. Under the intense competition of GLP-1, cyclopeptides may be driven away, but it will take time to get out of the cavity of DPP4. This will increase the interference time and influence the potential of cyclopeptides on the enzyme activity of DPP4.

Cyclopeptides play an important role in peptide drugs [47]. There are many studies on natural cyclopeptides such as antibiotics, antiviral, and anticancer, while there are relatively few studies on antioxidant, hypoglycemic, and antihypertensive effects, probably because some cyclopeptides come from toxic sources and are not suitable for general health care use. The three plants discussed in this article are known to be safe at recommended doses, are readily available, and have a high content of cyclopeptides. Although preliminary discussions on the potential of cyclopeptides in DPP4 inhibition are limited to docking studies, it suggests that a large number of Caryophyllaceae-Type Cyclopeptides (CTCs) may find new research topics in health care or nutritional supplementation. CTCs are only composed of amino acids without disulfide bonds, and some can be synthesized artificially. B Poojary et al. synthesized PB and discussed its antibacterial, antifungal, anti-inflammatory, and anthelmintic activities [65]. R Dahiya et al. designed coupling reactions of the tetrapeptide unit to synthesize DdC and indicated its antimicrobial and antihelmintic activity [66]. There are currently no reports on HB synthesis. Similar to DdC, HB has two glycines and two prolines that have the chance to induce β-turns and loop formation in the head-to-tail synthesis. In the tropics and remote areas, the properties of antimicrobial and anthelmintics may be the focus for *D. cordata*, and anti-inflammatory is its common application in folk records. HB has also been noted to improve inflammation by inhibiting the PI3K/Akt pathway [67]. Anti-inflammatory, an additional effect of phyto-cyclic peptides, may bring benefits to T2D as diabetic complications are often caused by long-term poor glycemic control and chronic inflammation [2]. The DPP4 inhibitor Linagliptin has been noted to reduce obesity-related inflammation and insulin resistance [68]. DdC, HB, and PB have anti-inflammatory, hypoglycemic, and synthesizable potentials and are attractive compounds for further research.

### 2.3. Linear Peptide ”IFGGLPPPP” as the Reference Coordinate of “IFGGLPPP” (HB Linear Precursor) Derivative

**Figure 7:**
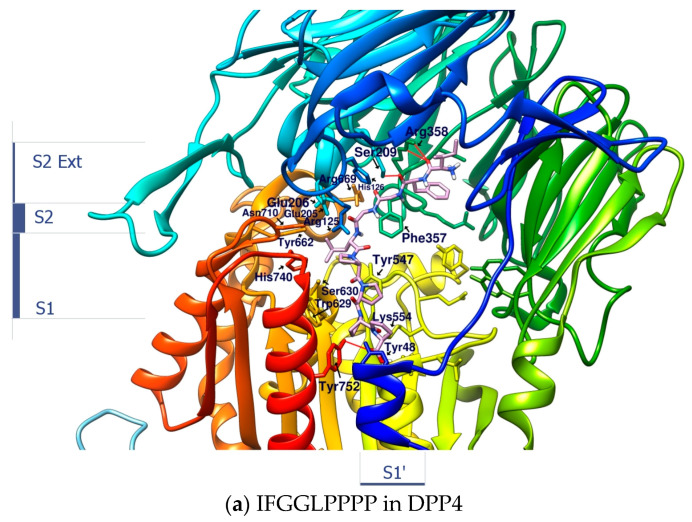

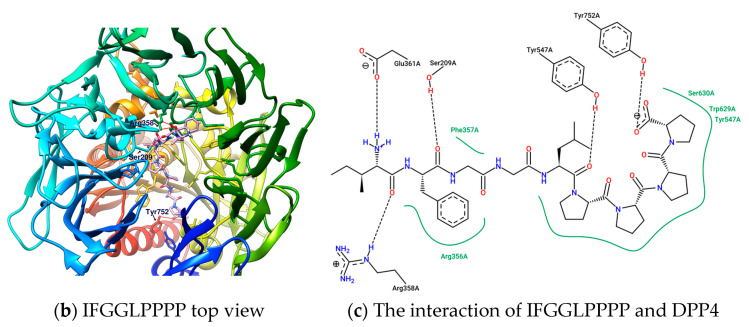
The configuration prediction and interaction analysis of IFGGLPPPP docking to DPP4.

Linear peptides may be quickly degraded under enzymatic hydrolysis, leading to loss of activity; however, the synthesis technology of linear peptides is much simpler than that of cyclopeptides. Their conformations in the liquid phase are more flexible and variable than cyclopeptides. This may bring benefits or disadvantages to the role of enzyme inhibitors. A series of linear peptides or protein hydrolysates that can survive after being hydrolyzed by gastrointestinal enzymes have been confirmed to show inhibitory effects on DPP4, but the discovery and verification process requires a lot of work. After docking the open-loop sequences “IFGGLPPP” and “GGPYWP” from HB and DdC with DPP4, the binding affinity of the two was found to be better than −9.0 kcal/mol. The configuration shows that the GGPY fragment of GGPYWP interacts with Arg125, Tyr456, and Tyr585. In contrast, the backbone of IFGGLPPP was more flexible, and its position on DPP4 was consistent with the appearance of most mainstream DPP4 inhibitors (from Ser209, Arg125 to Ser630). The predicted conformation of linear peptide IFGGLPPP in DPP4 was different from that of the cyclic HB. HB had cyclic constraints, directing its PPP residues to interact with the key amino acids on DPP4. How will linear peptides interact with DPP4 without the constraints of loops? By designing a series of IFGGLPPP derivatives (under the principle of avoiding DPP4 cleavage), it is possible to observe and compare their interaction with DPP4 and assess which ones have potential as DPP4 inhibitors. There is an example of the “PPPP” sequence in the proteolytic hypoglycemic peptide. After trying to dock IFGGLPPPP (nonapeptide) to DPP4, it was found that it extends from S2 Ext, S2, S1 to S1′ area (from the propeller entry to the side entry). It could be seen that the length of nine peptides was enough to occupy the interval where GLP-1 appeared in DPP4, as shown in Figure 7. It could also be observed that the IFGGLPPPP sequence did not penetrate the vicinity of the catalytic center such as His740, Asn710, and Try662. Introducing F or W residues into the “GGLP” sequence may be a way to add additional π–π interactions, thereby making the linkage of the peptide to the catalytic center tighter.

### 2.4. Design and Analysis of “IFGGLPPP” Derivatives as Potential DPP4 Inhibitors

**Figure 8:**
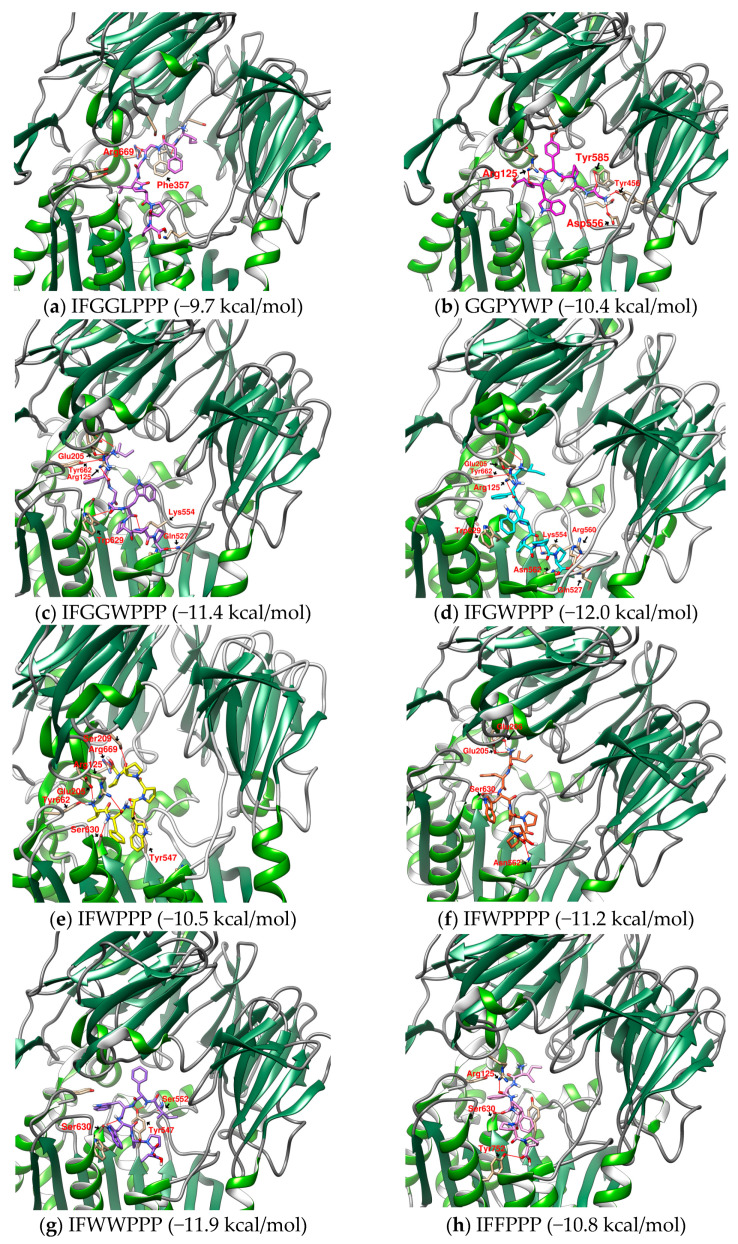
(**a**–**h**) Examples of IFGGLPPP-derived peptides as potential DPP4 inhibitors (UCSF Chimera 3D rendering with H-bonds labeling). Hydrogen bonds are shown as red lines and labeled with amino acid residues.

**Figure 9:**
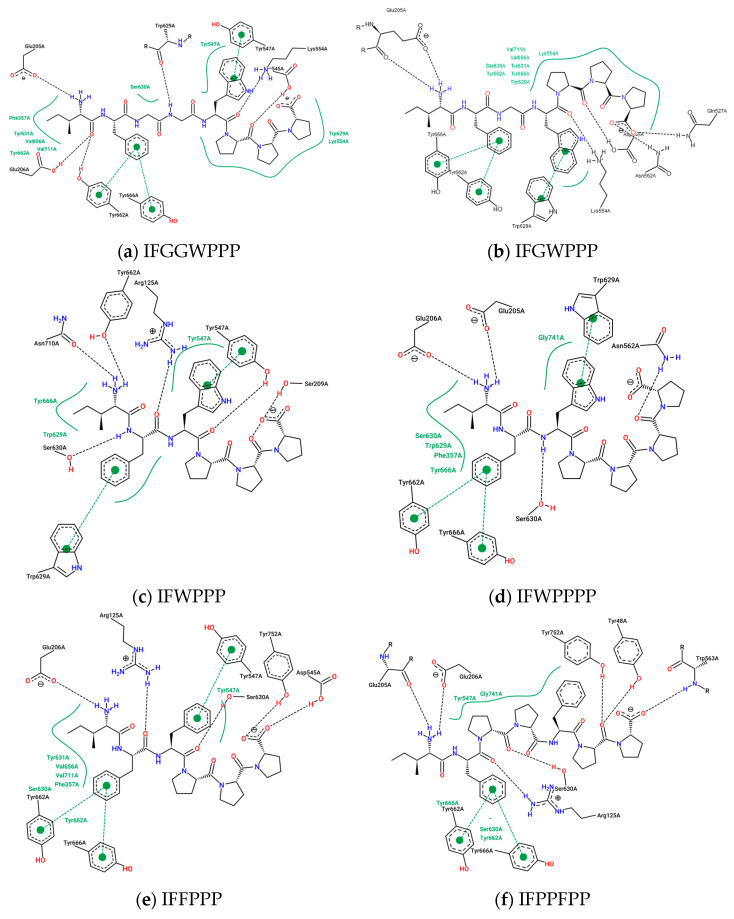
Interaction between the IFGGLPPP-derived peptides and DPP4 by PoseView.

In the discussion of DPP4 inhibitors derived from protein hydrolysates, there are short peptides such as IP and IPA as well as examples with longer sequences than IFGGLPPPP. When designing IFGGLPPP derivatives, what strategies should be adopted to expect peptides to block the entrance of the catalytic zone and interact with important amino acids? The results of the docking of IFGGLPPP derivatives with DPP4 are shown in Table 4. After docking IP to DPP4, it was found that there was a hydrogen bond between its Ile (I) and Tyr 662, but the binding affinity was only −6.6 kcal/mol. The docking affinity of IPA and IPI to DPP4 was −7.1 and −7.4 kcal/mol, respectively, which was better than the dipeptide. IFP, IFPP, and IFPPP are obtained by removing the middle part of IFGGLPPP. It was found that as the length of the sequence increased, the binding affinity also increased. If GL is replaced with W and F (longer length and possible π–π interaction), the results of the docking are more diverse. A series of linear peptides starting with IF and ending with PPP seem to have some common relationships with DPP4 (Figure 8 and Figure 9). The sequence of IFGWPPP, IFGGWPPP, IFFPPP, and IFWPPPP is created by replacing “GGL” of IFGGLPPP with GW, GGW, F, and WP. The Ile (I) at the beginning of the sequence establishes a key hydrogen bond with Glu205, followed by the π attraction between Phe (F) and Tyr662 and Tyr666, which become the basis for these peptides to associate with DPP4. Additionally, other possible π–π interactions between F/W and Try629 and Tyr547, or hydrogen bonds between PPP/PPPP and Ser630, Lys554, Asn562, Tyr752, etc., make these linear peptides (without secondary structure) seal the catalytic triad similar to a tape and produce a stronger effect than IP. This tape-like binding is the major interactive format between most IFGGLPPP derivatives and DPP4. IFFPPP (−10.8 kcal/mol), IFWPPPP (−11.2 kcal/mol), IFGGWPPP (−11.4 kcal/mol), and IFGWPPP (−12.0 kcal/mol) showed good binding affinity. Some of their interactions with DPP4 went beyond Lys554 (and even reached Arg560 and Gln527). Although this part had no direct effect on the catalytic region, it should provide substantial help for stabilizing the variable linear peptide structure on DPP4. The docking prediction showed that this functional “auxiliary PPP tail” had a quite different impact on DPP4 from PPP in HB. 

Short peptides ranging from tripeptides to tetrapeptides may be able to squeeze into the catalytic center region, thereby affecting the activity of DPP4. But such short peptides may also be easily pushed away by competition from GLP-1. The length of the 4–9 peptides may partly interact with amino acids near the catalytic center and partly serve to stabilize the structure. Longer peptides, however, may be at risk of degradation or low absorption. The docking score of multiple linear peptides was better than cyclopeptides. However, these are currently only reference values, and it is not clear which cyclic and linear peptides are more favorable for DPP4 inhibition under physiological conditions. The movement of cyclopeptides within the DPP4 cavity may interfere more with DPP4 activity than that of linear peptides. Furthermore, cyclopeptides generally have better bioavailability than linear peptides. 

Unlike the linear display of IFWPPPP, IFWPPP acts on DPP4 in a configuration similar to that of a cyclic peptide. In the IFGGLPPP-derived peptide series, the peptides suffixed with PPPP may obtain a better performance than PPP, but the increased P may also affect the configuration. The “IF” of IFWPPP goes deep between Asn710 and Ser630, where its “W” has a π–π interaction with Tyr547, and the end “PPP” goes up to Ser209 to form a C-shaped appearance. IFWPPP is the only example that interacts with Asn710 among all the cyclopeptides and linear peptides mentioned in this study. Compared with IFGWPPP (−12.0 kcal/mol), which had the highest score for linear peptide docking, IFWPPP (−10.5 kcal/mol) had weaker binding affinity; however, IFGWPPP partially acted on the region relatively far from the catalytic triad. In contrast, all the effects of IFWPPP have been focused on the key amino acids that classic DPP4 inhibitors are interested in. More substantial research is needed to determine which one is better. Similar to IFWPPP, IFWWPPP has a C-shaped conformation in the active region of DPP4. The C-shaped opening of IFWPPP faces the catalytic center, while the C-shaped opening of IFWWPPP faces outward. For IFWWPPP, the structure of two “W” in the sequence is more difficult to synthesize than one in IFWPPP. From the research on the docking of IFGGLPPP derivatives to DPP4, it was found that fragments obtained by cutting the cyclopeptides may be another way to create functional protein hydrolysates. These linear peptides fit DPP4 in a curved manner, possibly because they are derived from cyclopeptides or because the active area of DPP4 has a curvature. In addition, perhaps because the substrate of DPP4 is a peptide (GLP-1), this series of linear or cyclopeptides seem to have many interactions with DPP4 in docking. 

The docking of IFGGLPPP derivatives with DPP4 suggest that the method of extracting cyclopeptide sequences may bring a new option for the design of functional linear peptides. Molecular docking-recommended peptides can be further evaluated via subsequent in vitro/in vivo experiments. Later, peptides with developmental potential can be modified at the N, C-terminus, or specific molecules to make them more stable and effective in physiological environments [48]. Caryophyllaceae-Type Cyclopeptides provide hundreds of peptide sequences. If the cyclopeptide is freely cleaved, many peptide fragments can be obtained (e.g., IFGGLPPP, FGGLPPPI, LPPPIFGG, GGLPPPIF, IFGGLP, LPPPIF from Heterophyllin B, etc.). IFGGLPPP and GGLPPPIF behave differently in DPP4 space. They may also behave differently in different receptors. Docking these cyclic peptide-derived peptides to various drug targets may yield unexpected results. This may lead to new possibilities for drug design.

### 2.5. Molecular Dynamics Simulation of Potential Cyclic and Linear Peptides

**Figure 10:**
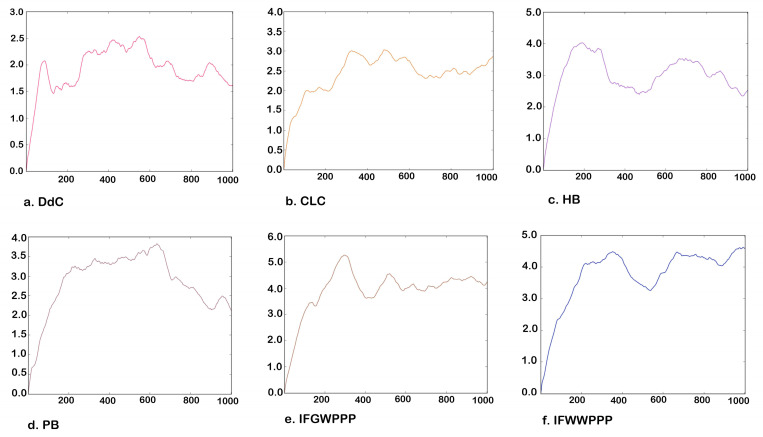
Molecular dynamics simulation of a series of peptides. Vertical axis: RMSD (Å). Horizontal axis: number of frames.

Further molecular dynamics simulations were performed on the lowest energy configuration (RMSD = 0) of the potential compounds. The calculated temperature was about 300 K. Under the dynamic observation of 1000 frames, the average RMSD of DdC, CLC, HB, and PB ranged from 1.6 Å to 2.5 Å, which is about a bond-length distance (Figure 10). MD simulation studies revealed stable binding throughout the simulation over 1000 frames, with small variation in configuration. Observing the changes in the configuration at 1000 frames, cyclopeptides can still maintain most of the interactions established with DPP4 at RMSD = 0, indicating the correctness of AutoDock Vina in the prediction. Among them, the DdC of small molecules had a relatively small offset and potential energy (Table 5). Compared with cyclopeptides, linear IFGWPPP and IFWWPPP have relatively large configuration changes and position shifts because linear peptides have a higher degree of freedom in structure. However, because IFGWPPP and IFWWPPP have a certain structure length, and the PPP tail has multiple interactions with DPP4, they still maintain a tape-like barrier to the exit of the catalytic zone, even with a relatively large average RMSD. The molecular dynamics simulation results were basically consistent with the prediction of the lowest energy configuration.

## 3. Materials and Methods

Molecular docking simulation places ligands in the binding site of receptors to find the configuration with the lowest binding energy. AutoDock Vina (Vina) is a program for molecular docking and virtual screening [70]. Vina uses an iterative local search global optimizer to provide high speed and accuracy of docking. The default setting of Vina is semi-flexible docking. During the docking process, the receptor is set to be rigid, while the ligand has a certain degree of freedom. When a large number of macromolecular ligands need to be evaluated, semi-flexible calculations provide a time-saving option. The study by Mishra A et al. titled “a Cyclic Octapeptide-Cyclosaplin from Sandalwood (cyclo-RLGDGCTR)” used Vina to screen a series of tumor-related receptors, which predicted that it could produce a stronger binding affinity with EGFR, VEGFR2, PKB, p38, etc. [71]. Y Hou et al. used Vina to demonstrate the possible conformation of Cyclo (PGFIPFTV) extracted from *Tunicyclin L*, acting on acetylcholinesterase (AChE) [72]. Z Wang et al. used Vina to analyze the interaction between natural Rubiaceae-type cyclopeptide (RA) and TAK1 protein to explain its possible involvement in the NF-κB pathway [73]. It was observed that AutoDock Vina is available for the evaluation of peptides as a variety of enzyme inhibitors, and may also be useful as an analysis tool to find potential DPP4 inhibitors.

The cyclopeptide structures (2D or 3D format) of *Pseudostellaria heterophylla, Linum usitatissimum, Drymaria diandra*, and related DPP4 inhibitors were downloaded from PubChem. The biologic description of plant cyclopeptides including molecular weight, IUPAC Condensed, PLN, etc., also came from PubChem and were organized into tables. MarvinSketch (ChemAxon), ACD/ChemSketch (ACD Labs), ChemDraw, and Avogadro were used as 2D and 3D molecular editing tools to produce structure formats that meet the requirements of docking software or to draw diagrams for explanations in the text. The initial structure of the linear peptide was established in Avogadro and went through an optimization process. The DPP4 crystal structure PDB: 3G0B used as the receptor in the docking was retrieved from the RCSB Protein Data Bank [60]. Before docking, the receptor and the molecule to be tested is executed in the “dock prep” procedure under UCSF Chimera 1.13.1 including “delete solvent”, “add hydrogens”, and “add charges”. Charges were computed using ANTECHAMBER [74,75]. The degrees of freedom of the ligands were automatically set during the preparation process. The prepared receptors and ligands were introduced into AutoDock Vina_1_1_2. The grid center was set to X = 42.049, Y = 34.288, Z = 14.618 (the centroid of the original ligand), and the grid size was set to 40 × 40 × 40. Vina automatically calculates the grid map. Then, the molecular docking program runs under the default settings. 

After the calculation, 10 sets of data within the maximum energy difference = 3 (kcal/mol) were obtained, and the lowest energy configuration with RMSD = 0 was selected as the analysis result. There may be very few exceptions when considering the most reasonable hydrogen bond interactions. For example, DmA’s result is displayed in the second energy level. The 3D structure image was rendered with UCSF Chimera, which contains the predicted configuration/conformation, receptor (DPP4), and hydrogen bond labeling. The search criteria for hydrogen bonds was “Relax constraints by 0.4 angstroms and 20.0 degrees” by the “FindHbond” program, and H-bonds are displayed in red lines. PoseView was used to further analyze the results of the interaction of IFGGLPPP derivatives with DPP4. It clearly depicted the relationship between the linear peptide and its surroundings including hydrogen bonds and π–π interactions. However, the cyclopeptide image produced by PoseView is not easy to read due to the overlap of the front and rear perspectives. BIOVIA Discovery Studio (DS) Visualizer was used to obtain the ligand-receptor 2D diagram with a circular format (DS does not show hydrogen atoms that have no special effect). The hydrogen bonds demonstrated by UCSF Chimera may be slightly different from those displayed by PoseView or DS because the conditions for analyzing the forces (default setting) from different software may not be exactly the same. The Peptide Analyzing Tool (Thermo Fisher Scientific) was used to calculate the molecular weight of linear peptides and confirm the possibility of synthesis/purification (theoretical pI of most linear peptides derived from IFGGLPPP is 6.0 with moderate hydrophobicity). Molecular dynamics were calculated through the functionality built into Chimera using the interface designed by V. Munoz-Roles and J.-D. Marechal. The setting conditions of the molecular dynamics simulation were “Steepest descent steps = 100; Steepest descent steps size = 0.02 Å. Conjugate gradient steps = 10; Conjugate gradient step size = 0.02 Å. Settings = Minimization. Start frame = 1; Step size = 4; ending frame = 1001; Lower RMSD threshold = 1.3; Upper RMSD threshold = 1.8”.

## 4. Conclusions 

A series of cyclopeptides from *Pseudostellaria heterophylla* (*P. heterophylla*), *Linum usitatissimum* (flaxseed), and *Drymaria diandra* have been reported to be beneficial in the treatment of diabetes. These cyclopeptides exhibited binding affinities by docking to DPP4 ranging from −8.4 to −10.7 kcal/mol. The binding affinity of 18 of the 25 cyclopeptides was better than −9.0 kcal/mol. It was found that the DPP4 inhibition may be a workable pharmacological target of these cyclopeptides. It also explains the possible reasons why these plants can be a dietary supplement or herbal medicine for lowering blood glucose. Docking showed that DdC, HB, DmA, CLA, CLB, CLC, and PB with two or more prolines in the sequence could obtain better binding affinity to DPP4 and have higher potential for further research. These cyclopeptides also happen to be abundant in the original plant. DdC (−10.7 kcal/mol) and HB (−10.4 kcal/mol) were the top two scorers, have an anti-inflammatory property, have the potential to be mass-produced, and are the most recommended molecules to be studied individually. In addition, various derivatives of HB linear precursor IFGGLPPP such as IFGGWPPP, IFGWPPP, and IFWPPP exhibit the potential for DPP4 inhibition. It was found that the introduction of W into the sequence of IFGGLPPP increased the interaction of the derivatized peptide with the vicinity of the catalytic center, and the PPP tail could facilitate the stabilization of the linear peptide in the active site. This brings new ideas for the design of functional linear peptides, especially for receptors with peptide ligands, curved structures, and large active sites. Further research is needed.

## Figures and Tables

**Table 1:** Cyclopeptides of *Pseudostellaria heterophylla* and the related binding affinity (BA) to DPP4.

N.	Abbr.	Compound	M.W. (g/mol)	Structure Name	BA (kcal/mol)
1	HA	Heterophyllin A	727.9	(cyclo)-PVIFGIT-(cyclo) [37]	−9.2
2	HB	Heterophyllin B	778.9	(cyclo)-GGLPPPIF-(cyclo)	−10.4
3	HC	Heterophyllin C	703.9	(cyclo)-GPIIPIL-(cyclo)	−8.8
4	HJ	Heterophyllin J	487.5	(cyclo)-AGPVY-(cyclo)	−8.8
5	PA	Pseudostellarin A	501.6	(cyclo)-AGPYL-(cyclo)	−8.4
6	PB	Pseudostellarin B	682.8	(cyclo)-GGGPPFGI-(cyclo)	−9.6
7	PC	Pseudostellarin C	813	(cyclo)-GTLPSPFL-(cyclo)	−8.5
8	PD	Pseudostellarin D	713.9	(cyclo)-GPLILGY-(cyclo)	−9.6
9	PE	Pseudostellarin E	878.1	(cyclo)-GPPLGPVIF-(cyclo)	−9.3
10	PH	Pseudostellarin H	861	(cyclo)-GTPTPLFF-(cyclo)	−9.4

**Table 2:** Cyclopeptides of *Linum usitatissimum* and the related binding affinity (BA) to DPP4.

N.	Abbr.	Compound	M.W. (g/mol)	Structure Name	BA (kcal/mol)
1	CLA	Cyclolinopeptide A	1040.3	(cyclo)-ILLPPFFLV-(cyclo)	−9.8
2	CLB	Cyclolinopeptide B	1058.4	(cyclo)-IMLIPPFFV-(cyclo)	−9.8
3	CLC	Cyclolinopeptide C	1074.4	(cyclo)-IM(O)LIPPFFV-(cyclo)	−10.0
4	CLD	Cyclolinopeptide D	1064.3	(cyclo)-LLPFFWIM(O)-(cyclo)	−8.7
5	CLE	Cyclolinopeptide E	977.3	(cyclo)-IM(O)LVFPLF-(cyclo)	−9.1
6	CLF	Cyclolinopeptide F	1084.4	(cyclo)-LM(O)PFFWVM(O)-(cyclo)	−9.3
7	CLG	Cyclolinopeptide G	1098.4	(cyclo)-LM(O)PFFWIM(O)-(cyclo)	−9.3
8	CLH	Cyclolinopeptide H	1082.4	(cyclo)-LMPFFWIM(O)-(cyclo)	−9.1
9	CLI	Cyclolinopeptide I	1068.4	(cyclo)-LM(O)PFFWVM-(cyclo)	−9.2
10	CLJ	Cyclolinopeptide J	993.3	(cyclo)-IM(O_2_)LVFPLF-(cyclo)	−9.1

**Table 3:** Cyclopeptides of *Drymaria diandra* and the related binding affinity (BA) to DPP4.

N.	Abbr.	Compound	M.W. (g/mol)	Structure Name	BA (kcal/mol)
1	DmA	Drymarin A	1016.2	(cyclo)-AFPPPFFVI-(cyclo)	−10.2
2	DmB	Drymarin B	674.8	(cyclo)-GLPFYP-(cyclo)	−8.9
3	DdA	Diandrine A	747.8	(cyclo)-GPWPYF-(cyclo)	−9.6
4	DdB	Diandrine B	838	(cyclo)-GPLPLWSS-(cyclo)	−8.9
5	DdC	Diandrine C	657.7	(cyclo)-GGPYWP-(cyclo)	−10.7

**Table 4:** Results of the docking of IFGGLPPP derivatives with DPP4.

N	Sequence	S2 Ext	S2 and S1 Pocket	S1′ and Surrounding	Periphery	BA (kcal/mol)	MV (g/mol)
1	IFGGLPPP	F357 F357π	R669	Y547	-	−9.7	796.9733
2	IFGGLPPPP	S209, R358 E361	-	Y547	Y752	−10.3	894.0913
3	GGLPPPIF	-	E205, S630, W629π	Y547, K554	-	−8.9	796.9733
4	IP	-	Y662	-	-	−6.6	228.2914
5	IFP	-	S630, Y662π, Y666π	-	-	−8.4	375.4680
6	IFPP	-	W629, S630, Y662π, Y666π	Y547	-	−9.5	472.5903
7	IFPPP	-	E205, Y662π, Y666π	Y547	-	−10.0	569.7083
8	IFPPPP	-	R125, S630, Y662π, Y666π	D545	Y752	−10.4	666.8263
9	IFFPPP	-	R125, E206, S630, Y666π	D545, Y547π	Y752	−10.8	716.8863
10	IWWPP	F357π	E206, S630, W629	D545, Y547, C551	-	−11.1	697.8423
11	IFWPP	-	R125, Y666	Y547, Y547π	-	−9.8	658.8053
12	IFWPPP	S209	R125, S630, N710, Y662, W629π	Y547, Y547π	-	−10.5	755.9233
13	IFWPPPP	-	E205, E206, S630, W629π, Y662π, Y666π	N562	-	−11.2	853.0413
14	IFWWPPP	F357π	S630, Y662π, W629π	Y547, C551		−11.9	941.4800
15	IFGPPPP	-	R125, E205, Y662, Y662π, Y666π	D545, K554	-	−10.7	723.8783
16	IFGWPPP	-	E205, Y662π, Y666π, W629π	Q527, K554, N562	-	−12.0	812.9753
17	IFGGWPPP	-	E206, Y662π, Y666π	D545, Y547, K554	-	−11.4	870.0273
18	IFYWPPPP	-	R125, W629π	V546, K554	Y752 Y48	−11.3	1016.219
19	IFPPFPP	-	R125, E205, E206 S630, Y662π, Y666π	W563	Y752 Y48	−10.6	814.0043
20	IFYGPPP	-	E205, E206, S630 Y662, Y662π, Y666π	V546, K554 Y547π, K554	Y752	−10.4	789.9383
21	IFIFPPP	-	R125, E205, S630 Y662π, Y666π	-	Y752	−10.0	716.8863
22	GGPYWP	F357π	E206	Y456, Y547π D556, Y585	-	−10.4	675.7483

(a) The division of S1, S2, S1′, S2 Ext refers to related studies by Yoshida, T. et al.; Berger, JP et al.; and Arulmozhiraja, S et al. [18,19,69]. S1 and S2 pockets include W629, S630, N710, H740, R125, E205, E206, Y662, Y666, and R669 (S1 pockets generally refer to S630, N710, H740, W629, Y662, and Y666. S2 pockets refer to E205, E206, and R125. These amino acids are difficult to distinguish in some selected perspectives for 3D rendering. In the table, the ranges of S1 and S2 are listed in the same column). S2 extensive sub-site (S2 Ext) and surrounding include V207, S209, F357, R358, and E361. S1′ and surrounding includes D545, V546, Y547, Q553, K554, N562, Y585, etc. Periphery includes Y752, and Y48 near S1′. (b) When marked as R125, E205, S630, etc. in the table, it indicates that there is a “hydrogen bond” between the designed peptide molecule and the amino acid of DPP4. (c) When there is an additional π tag such as F357π, W629π, Y662π, etc., it indicates that there is a “π–π interaction” with the amino acid of DPP4. The above interaction analysis comes from the analysis of PoseView.

**Table 5:** Average RMSD and energy (KJ) obtained from molecular dynamics simulations.

	DdC	CLC	HB	PB	IFGWPPP	IFWWPPP
Number of atoms	48	76	56	49	59	69
Average RMSD over 1000 frames	1.611	2.43	2.514	2.118	4.214	4.582
Average Potential energy	747.162	987.740	931.417	792.090	955.261	938.587
Average Kinetic energy	336.549	628.665	472.127	342.365	430.523	486.235

## Data Availability

Data are contained within the article.
